# Undergraduate nursing and medical students’ perceptions of food security and access to healthy food in Qatar: a photovoice study

**DOI:** 10.1017/jns.2022.28

**Published:** 2022-04-29

**Authors:** Areej Al-Hamad, Shannan MacNevin, Suhad Daher-Nashif

**Affiliations:** 1 Faculty of Nursing, University of Calgary in Qatar, Doha, Qatar; 2 College of Medicine, Qatar University, Doha, Qatar

**Keywords:** Food security, Healthy food, Photovoice, Undergraduate students

## Abstract

The present study explored nursing and medical students’ perceptions of food security, their access to healthy food and the circumstances that affect their access to healthy food in Qatar. The photovoice method was adopted in the present study. Students submitted their photos pertaining to food security and their access to healthy food in Qatar. Afterwards, the students completed an online synchronous semi-structured interview. The interviews were transcribed verbatim and thematically analysed. After the data analysis, a focus group discussion was conducted for member checking. The present study is a collaborative project between two universities in Qatar: The University of Calgary in Qatar (UCQ) and Qatar University (QU). Undergraduate students (seven nursing students and nine medical students) were recruited, asked to collect photos and interviewed. Four themes emerged from the data. First, food retail environments promoted unhealthy eating. Second, fast food under stressful circumstances: a sense of comfort. Third, food as a symbol of culture and socialisation. Finally, the paradox of access to affordable and healthy food in Qatar. Undergraduate students highlighted various circumstances that affect their perceptions of food security and their access to healthy food in Qatar. Future research that aims at understanding the facilitators and barriers to access healthy food at the university campus may help to improve nutrition interventions targeting those students. Future initiatives should focus on leveraging various resources to assist universities in tailoring their food initiatives to suit their students’ local needs.

## Introduction

Food security is more than a necessity for well-being; it is a human right that affects global safety, economic strength, security and sustainability^(1,2)^. Food security is defined as ‘a situation that exists when all people, at all times, have physical, social and economic access to sufficient, safe and nutritious food that meets their dietary needs and food preferences for an active and healthy life’^(3)^. Food security is characterised by four dimensions, including availability, accessibility, utilisation and stability^(4)^. These dimensions are closely related and the neglect of any of them would adversely affect food security. Key to understanding the role of food security in promoting population health are clear definitions of the pillars of food security and their appropriate contextualisation. The Food and Agriculture Organization (FAO) defined food availability as the ‘physical existence of food and on national level it involves a combination of domestic food production, food imports and exports, and domestic food stocks’^(3)^. Food accessibility refers to food affordability, allocation and preferences^(5)^. The use of food to provide sufficient energy and nutrients is the main aspect of food utilisation whereas food stability is often connected with the time frame during which food remains constantly available during various times of the year^(3)^. To the best of our knowledge, there are no clear definitions of food security and its related dimensions that emphasise the unique nature of such issues within a Qatari context.

Food security in Qatar is a multifaceted issue that is recognised by several government stakeholders, which requires action across sectors to be achieved and maintained. In Qatar, meeting the population's demand for food is challenging because it relies mostly on available domestic food supplies since the geopolitical blockade in June 2018^(6)^. There are several factors that may challenge the achievement of food security in Qatar, including water crisis, soil fertility and the adverse effects of harsh geoclimatic conditions^(6,7)^. Collectively, these factors increase the burden and pressure on Qatar's entire agricultural system and the capacity of the land, forcing it to import most of its food needs^(6)^. Recognising the importance of food security for a healthy and thriving population, and despite all the aforementioned challenges, Qatar's government has developed various strategies to improve food security among its residents during the geopolitical blockade on Qatar that was started in June 2018 and was removed in 2021. These strategies include establishing local companies that are producing poultry and dairy products^(6,7)^.

Global Food Security Index (GFSI) addresses various issues of food affordability, accessibility availability, quality and safety, and natural resources and resilience across a set of around 113 countries through a dynamic benchmarking model constructed that measure different drivers of food security across countries^(8)^. In a recent local media report, and with regard to the GFSI, it was highlighted that Qatar ranks first among the Arab countries; however, it has decreased in terms of ranking from occupying the 22nd place to the 13th place globally^(6,7)^. This achievement in Qatar's food security highlights the efforts being made in opening local markets in Al Khor, Al Thukhaira, Al Wakra, Al Shamal and Al Shahaniya^(9)^. Despite the notable achievements made by Qatar's government, there is a paucity of research in Qatar on undergraduate students’ food security and access to healthy foods. Several studies have been conducted to explore food security among undergraduate students that touched upon healthy eating behaviours, obstacles to get food at the university campus and levels of food security among university students^(10–12)^. To the best of our knowledge, no studies about food security have been conducted among undergraduate university students in Qatar or the Gulf region.

Nursing and medical students have typically been overlooked as an at-risk population in regards to food security and their eating behaviour; the reasons for the same are their busy schedules and heavy workload^(11)^. Nursing and medical students interact with various groups of the population who might be struggling to achieve food security or access to food on a daily basis^(13)^. Therefore, they should be made well aware of the concept of food security, access to healthy foods and the different dimensions of food security, as these are significant determiners of health for the populations whom they deal with or treat in the future. Moreover, it is one of the responsibilities of these students as future healthcare providers to be advocates of food security since this is a vital factor for leading a healthy lifestyle^(14)^.

This research is aligned with the Qatar National Research Fund (QNRF) and with Qatar's national food security strategy for the period 2019–2023. The executive director of QNRF has identified the different environmental, climatic and political aspects of food security as one of the major research priorities for Qatar^(9)^. The food security strategy aims at fostering the development of productive and sustainable food systems, at enhancing food security measures, and at improving a healthy lifestyle in Qatar through investing in applied research^(6)^. This research project also aligns with the directives propounded by Emir of Qatar for self-reliance and self-sufficiency in the production of food and medicine, the diversification of resources and the attainment of economic independence^(6)^.

The present study is the first to explore undergraduate nursing and medical students’ perceptions of food security and the circumstances that affect their access to healthy food in Qatar. An exploration of nursing and medical students’ perceptions about food security and the factors influencing their access to healthy food in Qatar is required for advocacy, policy development and strategies to enhance the health of the people of Qatar. The study findings will fill the gaps in knowledge^(6,9)^ and will help to inform policy development and changes regarding food security and access to healthy food in Qatar. We anticipate that the present study will have several benefits for undergraduate healthcare students in Qatar, including: (1) promoting an awareness on the importance of food security as an integral part of a healthy lifestyle; (2) emphasising the importance of productive collaboration between different healthcare specialities for enhancing food security; (3) encouraging undergraduate students to pursue graduate-school education by offering them hands-on training on various processes of scientific research; and (4) offering an opportunity for undergraduate students to conduct more refined and collaborative projects by building team-based research collaboration with faculty and mentors.

## Methods

This research study is a collaborative joint project between two universities in Qatar: the UCQ and QU. The present study used the photovoice method, a community-based participatory action research approach that is commonly used in health research. This method is rooted in the principles of the critical consciousness theory^(15)^, the feminist theory and documentary photography as a means to bring about social change and reform^(16)^. This method requires individuals to take and use photographs as a visual representation to identify and address pressing issues in their community^(16,17)^. The uniqueness of the photovoice method is that it allows participants to take photographs, gives a ‘voice via camera’^(18)^ to a particular group and uses photos as a vehicle to reflect their perceptions and to communicate these issues to policymakers and stakeholders from the participant's point of view^(16,17)^.

Photovoice is a research approach that ensures that everyone's voice is heard to reflect collective experiences by implementing the mnemonic VOICE (Voicing Our Individual and Collective Experience)^(16,17)^. Recently, photovoice has been used in several research studies with food security, youth and university students^(19–22)^. Many researchers have asserted that photovoice minimises the power differential between researchers and participants by granting autonomy to the participants and encourages group participation, creativity and self-reflection^(17,19,23,24)^. Previous studies have shown that the photovoice method is valued by college students and offers an interactive atmosphere for them to engage in, have fun, be empowered and think beyond the personal level in the research process^(18,25)^. Photovoice has interesting features, including a participatory means of sharing experiences, a tool for social change, and providing greater insights than traditional research methods^(10,18,24,25)^. This study was conducted according to the guidelines laid down in the Declaration of Helsinki, and all procedures involving research study participants were approved by the following ethics boards: (1) University of Calgary's Conjoint Health Research Ethics Board (REB20-1972), (2) QU-IRB (QU-IRB 1514-EA/21) and (3) IEC PHCC (PHCC/DCR/2020/12/142). Written informed consent was obtained from all subjects.

To ensure the security, confidentiality and anonymity of the participants’ data and pictures, several measures were taken. First, anonymous labels and identifiers were used on all hard copy and electronic data to mitigate the risk of identification. Second, all paper documents and pictures were stored in locked filing cabinets in the research department at UCQ, where they would not be accessible to anyone except the research team. The participants were advised to load their collected photos onto laptops and send them to their primary mentor through their university email to ensure safety and security. Photographs and electronic files were stored on an encrypted password-protected computer belonging to the research team. The computer was stored in a locked office at UCQ. Hard copies, pictures and electronic data would be destroyed by mechanical shredding 5 years after completion of the study.

### Procedures

A purposeful sample of sixteen undergraduate students (seven nursing students and nine medical students) from the two universities was considered for the present study. A review of the literature showed that it supports a sample size of fifteen to twenty participants to conduct photovoice projects^(19,20,22,24)^. This sample size of students ensured a fair and representative sample from both settings to capture students’ perceptions across the two universities, including the different genders of students. Study participants received training about the photovoice methods, including the ethical considerations of photo taking. The participants received iPads from the participating universities, and this enabled the students to photograph their perceptions of food security and the circumstances that affect their access to healthy food in Qatar. For instance, students were asked to take photos of their environment and the places linked to the purpose of the study, such as home, university, grocery shops or markets and parks. The study participants were given 2 weeks to collect pictures.

After 2 weeks and due to the COVID-19 pandemic, the research team (undergraduate research assistants) conducted a 45 to 60 min online synchronous semi-structured individual interview by using a university-secured version of Zoom platform. A semi-structured interview guide based on the mnemonic **SHOWeD** developed by Wang(^16,17)^ was used to allow the participants to describe each photo that they had taken. **SHOWeD** stands for the following questions: What do you **SEE**? What is really **HAPPENING**? How does it relate to **OUR** lives? **WHY** does the situation exist? What can we **DO** about it? All the interviews were digitally recorded.

### Data analysis

All the digitally recorded interviews were transcribed verbatim into a word document. Data were entered and analysed by using NVivo software version 12^(26)^. Data were thematically analysed by using an iterative, inductive approach^(27)^. The research team was fully immersed in the data on reading the transcribed interviews several times with an in-depth engagement and extensive coding. Initially, a codebook was developed for each participant; three researchers from the team reviewed and independently analysed all the transcripts and photos to revise or refine the coding. All discrepancies were discussed and resolved by the research team. Finally, a focus group discussion session was held with the interested participants to validate the study findings and to enhance the credibility of the analysed data. The research team asked the participants to select the final pictures to be considered for the photo exhibits while presenting the research findings.

## Results

A total of sixteen undergraduate students (seven nursing and nine medical students) participated in the photovoice study. The participants’ age ranged from 18 to 32 years; fifteen were female, and one was male. Five students identified as Qatari and eleven were non-Qatari, that is, they belonged to different nationalities. All the study participants were single, and two of them held part-time jobs. The participants’ years of study ranged from 1 to 4 years. Four primary themes emerged from the students’ photos and interviews: (1) Food retail environments promoted unhealthy eating; (2) fast food under stress: a sense of comfort; (3) food as a symbol of culture and socialisation; and (4) the paradox of access to affordable healthy food.

### Theme 1: Food retail environments promoted unhealthy eating

**Fig. 1.**
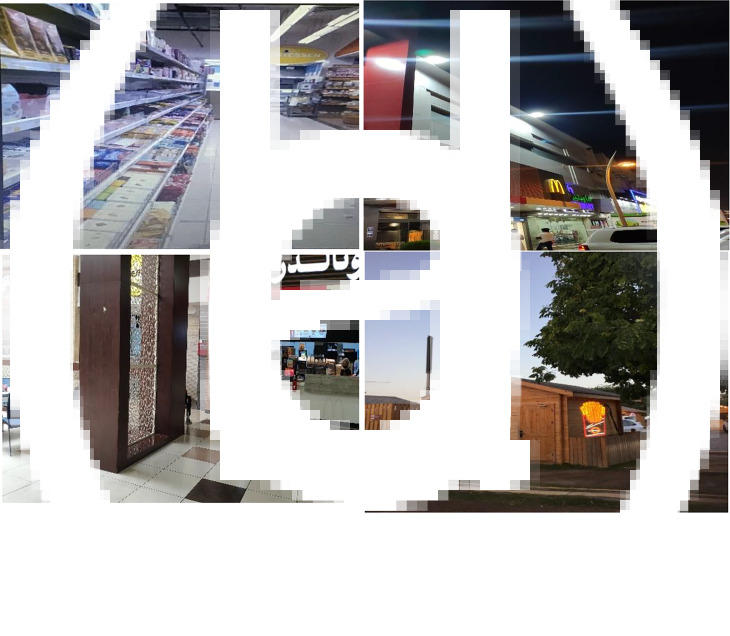
Photos depicting the theme of food retail environments that promoted unhealthy eating: (a) the main entrance of a grocery store presenting candies, chocolate bars and sugary items; (b) and (c) fast-food outlets inside a large grocery store and (d) only fast-food outlets available around the museum.

Students described how the nature and context pertaining to the food environment promoted their unhealthy eating behaviours. Photos were taken from a variety of convenience stores, grocery stores and food outlets and these reflected the messaging around the distribution and availability of unhealthy foods that encouraged unhealthy eating. In Fig. 1(a), the photo depicts the impact of the structure and layout of the grocery stores on buying unhealthy items, where the first aisle inside a large grocery store showed a huge collection of chocolates and candies. The student commented, ‘the grocery stores are laid out in a way that the first thing I see are chocolates and guess what's on the last aisle vegetables and fruits and how that can have an impact on what I will buy or eat’.

In Fig. 1(b) and (c), the photos illustrate how the distribution of fast-food outlets, their location and their competitive prices with frequent promotions affect students’ food intake. The student who took the photo said, ‘Sometimes we do go to McDonald's before we had our grocery, we went to McDonald's first, you know it is the first thing in the food court and to gain some energy for running and walking around the grocery, because it's cheap and has a lot of promotion’. Another student described the effect of competitive prices and the promotion of fast food compared with the limited promotion of healthy food on their eating or shopping behaviours: ‘the meal was 26 riyals in total which is not a lot I think for two people at all. I use the McDonalds app so you get points every time you make a purchase and then you can buy food with the points that you have, I think it might have been because it says promo at the top’. As depicted in Fig. 1(d), a student took a picture of a fast-food outlet as it was the only available food option around the museum where they used to go during the weekend or for their family gatherings. Students expressed their desire to have various food options, including healthy food, at public spaces such as parks and museums.

### Theme 2: Fast food under stress: a sense of comfort

**Fig. 2.**
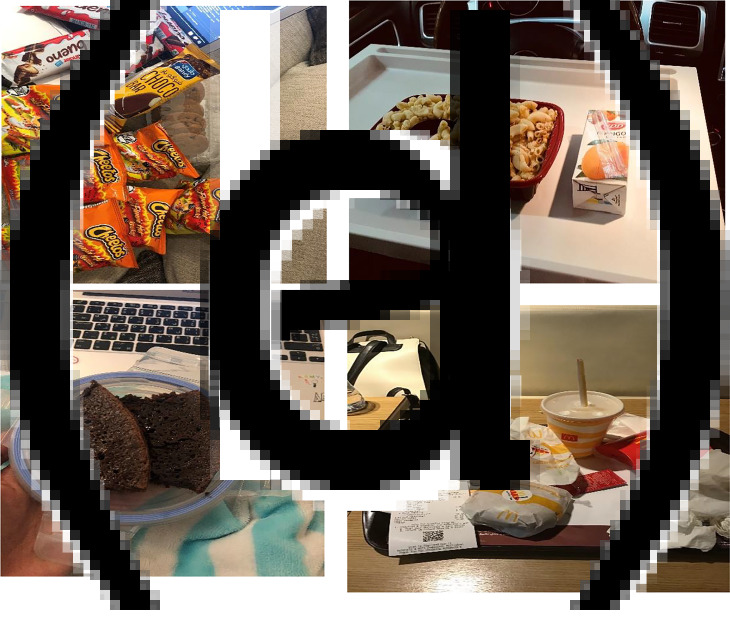
Photos depicting the theme of fast food under stress: a sense of comfort: (a) unhealthy snacks during exams; (b) eating a bowel of pasta and drinking juice in the car during clinical days; (c) a piece of cake to satisfy hunger during online classes and (d) a fast-food meal for a long day at the university campus.

Students described how fast food is an easy option to consider during stressful times, such as on exam days or clinical days. In Fig. 2(a) and (c), the photos depict the unhealthy snacks and chocolate bars that students usually eat to satisfy their hunger during exam days or during online classes, where there is no time to cook and prepare healthy food. A student commented, ‘as a nursing student I don't really have the time to prepare food to eat for the purpose of nutrition, I just eat to satisfy my hunger. You know chocolates and chips are cheap, so convenient and can subside your hunger. Being a student is sort of what food you have or quick to prepare or to cook’. The student also stressed that their clinical placement shifts with no time to prepare food or healthy snacks forced them to rely on ordering fast food to eat during rush and busy clinical shifts.

In Fig. 2(b) and (d), the photos depict how students usually rely on fast food and eating in a car, during their clinical shifts, to satisfy their hunger without paying heed to the nutritional value of the food they consume. A student reported, ‘like most of our colleagues, whenever we're in clinics, we eat or take away food and soda, most of the time when you're really hungry and just too lazy to make food you just want to get something to eat, you just eat it, you don't look at the nutrition label, you need something fast and ready, it's easier just to go to order at a fast-food restaurant and get a meal’. Another student stated that most of the times students skip their breakfast due to a lack of time, laziness and their clinical shifts or classes starting early. The student commented, ‘during my morning shift, it is difficult for me to wake up that early and at the same time to prepare breakfast and food that will last me the entire morning shift. Most of the time, I haven't had like breakfast other than water, so I haven't had any solid food, so I feel like the need to eat pasta, a plate of pasta, to have the energy for my shift’.

### Theme 3: Food as a symbol of culture and socialisation

**Fig. 3.**
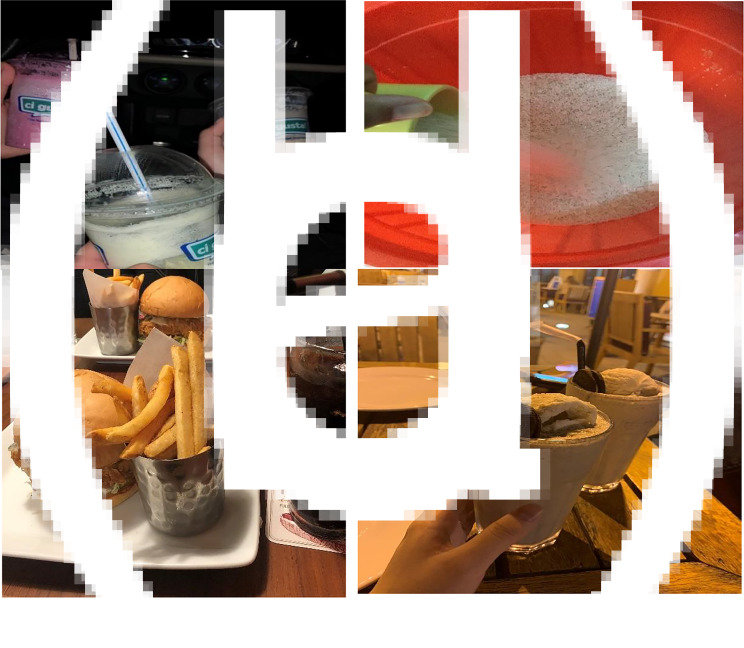
Photos depicting the theme of food as a symbol of culture and socialisation: (a) having smoothies with friends; (b) rice is a basic ingredient of our cultural food; (c) socialising and enjoying a meal together and (d) having ice cream with friends.

Students described that eating is a key for students’ socialisation, where they can have fun and celebrate their success or course completion. In Fig. 3(a), (c) and (d), a student reported that sometimes having food together is not for food *per se*, rather it is for socialising and spending time together at the university campus or clinic. One student commented, ‘eating food together, I think it's not like eating fast food is somewhat a family or friends gathering thing, we myself and a friend of mine love to go out together and eating together after we finish exams’. Another student remarked and echoed the same idea of food for socialisation and stated, ‘eating out is not really something that is done every single day, but it's so normal to be with your friends and then you're like “oh we're hungry let's just order food and have fun”’.

An interesting finding is depicted in the photo in Fig. 3(b), which is a photo of rice taken by a student; rice reflects their cultural food and also indicates how their culture predominantly depends on rice. The student commented, ‘here a picture of rice, rice is our favourite, delicious and cultural food, our meals basically have some sort of rice with different spices, chicken or meat’.

### Theme 4: The paradox of access to affordable healthy food

**Fig. 4.**
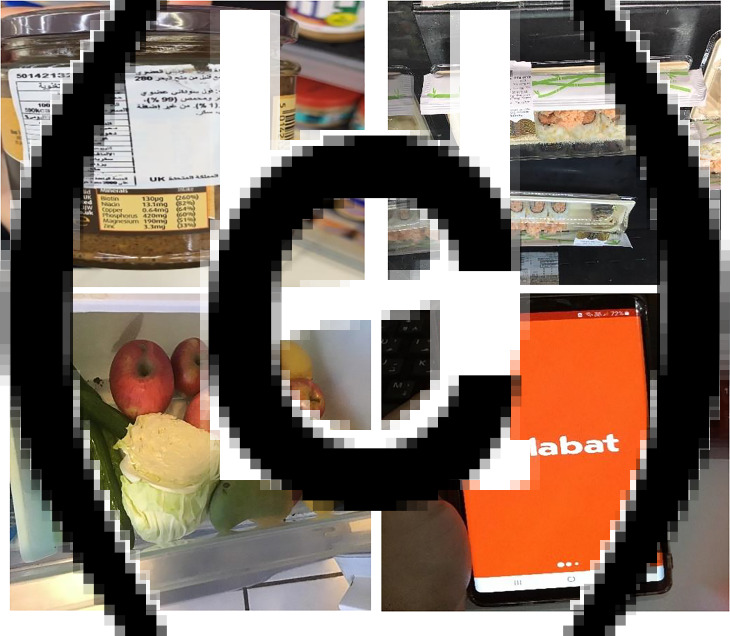
Photos depicting the theme of the paradox of access to affordable healthy food: (a) healthy and organic peanut butter imported from the UK at a reasonable price; (b) healthy sushi meal for QAR 10 only; (c) fresh fruits and vegetables are affordable and accessible and (d) Talabat is a friendly use online application to deliver food items.

Students assured themselves that they have access to healthy food at affordable prices in Qatar; they clearly stated that they feel they are food secure. On the other hand, students expressed a strong feeling related to the paradox of food access when it comes to having healthy food at university campuses or relying on vending machines or Talabat to order food, as depicted in Fig. 4(d). One student commented, ‘Food security for me, it means having a safe access to healthy food. I really feel that most of us have the food security here in Qatar, however, in the university if the cafeteria is closed or, you know, after lunch time and after the lunch hour rush, there aren't that many options left. If I get hungry, I just go with the flow, order food using Talabat application, so if I don't have food, I will eat what available in the vending machine’.

In Fig. 4(a), the photo illustrates the dichotomy of having access to healthy foods that are imported from abroad and that are simultaneously affordable at a reasonable price but not accessible through vending machines at the university. One student commented, ‘Also, there is a whole section for British imports, like food that is imported from UK like this organic peanut butter so I can buy food from the UK with no need to order thru Amazon and it is even cheaper. Again, it is in grocery store not university campus or vending machines food’. Another student remarked on the access and quality of food at university campuses. The student reported, ‘In between classes I didn't really have the time to sit and eat, I would go to the vending machine and get coffee and get like a packet of chips or whatever, this is the available and accessible option here, the quality of food in vending machines, it's all snack food really. When it comes to food, I think as university students, we always eat unhealthy food not because we are not aware of healthy food but because it's accessible and affordable’.

In Fig. 4(b) and (c), the photos reflect how healthy foods, including fresh vegetables and fruits, are available, accessible and affordable at reasonable prices in Qatar. One student reported that even some healthy foods, such as sushi, are ready-made and available at cheap prices. One student commented, ‘in some grocery stores, they actually have healthy and ready-made foods available. This was sushi, I think it's six pieces of sushi and it was 10 riyals which is quite cheap, considering its sushi’.

## Discussion

Using the photovoice method, the present study aims at exploring undergraduate nursing and medical students’ perceptions of food security and the circumstances that affect their access to healthy food in Qatar. Many studies have found associations between food security and diet-related outcomes, including academic performance^(28)^, diet quality^(29)^, food literacy and integrating food courses into the curriculum for nursing students^(30,31)^, effect of the COVID-19 pandemic^(32)^ and the impact of cultural food on the identity and well-being of undergraduate students^(33)^. The results of the current study assist in exploring these aspects by understanding how undergraduate nursing and medical students experience food security, their access to healthy food in Qatar and the various circumstances that affect their access to healthy food.

The first theme deals with students discussing the effect of the food retail structures and layouts to enforce the eating of unhealthy food. Foods that were unhealthy, sugary and high in calories were easily accessible at competitive prices and offers. In addition, the layout of grocery stores encourages unhealthy shopping behaviours as these stores place the chocolate and candy sections at the main entrance of the store. Conversely, fresh fruits, vegetables and other healthy items are placed far from the entrance or out of reach, and these are usually on limited offers. It could be argued that when shopping at retail stores, healthy ‘buying’ is a matter of choice, regardless of what is being displayed at store entrance or store layout. The study participants also emphasise that at some places the only available options for food are the fast-food retail stores where you will have no choice to find a healthy item to eat. These findings highlight the crucial factors that affect students’ healthy eating patterns, which include but are not limited to the arrangement of food sections, the location and access at grocery stores, supermarkets and other food stores. This finding is consistent with the study finding that concluded that the food environment has an essential role to play in the food shopping behaviours of university students^(34)^.

Participants also highlighted the effect of marketing strategies and signages, with frequent and competitive offers targeting unhealthy foods and beverages in the large grocery shopping centres, on their eating patterns. They also commented on the impact of the food court placement/location within the store layouts and on how the layout can encourage eating unhealthy food, especially when they reported being hungry and having no time to reach food stores or for buying healthy food items. These factors are congruent with previous studies^(35–37)^, depicting how the food retail environment affects adults’ food choices. Students described these factors with frustration, demonstrating how the food retail structures, layout and environment forced them to eat unhealthy food and prevented them from making healthier choices despite their desire and willingness to eat healthier food.

The second theme represents how fast-food options are common eating options for students during times of stress, including exams, online classes or clinical placement days, and how students perceived having fast food during these times as it provided them with a sense of comfort. These findings are consistent with prior studies, depicting how university students tend to rely on fast-food options during stress or exams^(38–40)^. Participants described that fast food, including chocolate bars or candies, are easily available options at reasonable prices and they did not require cooking or preparation, especially during exams or clinical placements where there is no time to cook or prepare healthy food. These findings are consistent with prior studies that showed that several factors affect university students’ desire to eat fast food and not to choose healthy food options^(34,39–41)^.

The third theme describes students’ perceptions of food as a symbol of culture and socialisation. As previously reported, adults’ personal attitudes and beliefs about food or food options are largely shaped and influenced by their socialisation patterns and they are a product of their culture^(42)^. Students reported that their food or food choices were intimately tied to their cultural food, which resulted from socialising with others, including their peers or their families. A noted aspect of this theme is that students frequently reported that their decision to eat and socialise is always tied to the effect of media on food promotions, regardless of the health impact or the healthy choices made. This finding is consistent with a study that showed the impact of the mass media on adolescent socialisation within the context of making unhealthy food choices^(43)^.

The fourth theme illustrates the paradox around the access to healthy affordable food available at reasonable prices in Qatar. Students perceived that they have access to affordable and healthy food in Qatar; however, many circumstances may affect their choice for healthy food, including a lack of time to cook or prepare food during exams or during stressful times. Another aspect that students emphasised is that healthy food is available, affordable and accessible in Qatar, but it is not necessarily accessible from vending machines across university campuses. This finding is congruent with other studies that concluded that universities should improve the nutritional value and quality of the food and beverages on their campuses’ vending machines that are accessible and affordable to students^(44,45)^.

The study findings may inform future practices and policies as well as they may strengthen the research culture in Qatar. Furthermore, these findings can inform the development of university-based food assistance policies and initiatives to better support university students with alleviating food insecurity. As described by the students in the present study, programmes or initiatives that promote healthy food availability or the provision of subsidies for healthy meals, fruits and vegetables at university campuses can encourage students to eat healthily. Given the strong impact of food quality on students’ mental and physical health and academic performance, healthy food awareness campaigns or programmes should be incorporated into the student's curriculum and food programmes to holistically address university students’ food security and its consequences. From a policy perspective, developing food policies for university campuses that calls for initiatives and strategies to improve the access, availability, affordability and price of healthy foods is of significance and would encourage healthier food choices^(46)^.

For data dissemination and to support knowledge mobilisation, a photo exhibit was held at the UCQ campus, and invitations were sent out to interested community members. The selected pictures were printed, laminated, hung and viewed by the invited members to offer an opportunity to show the message of the participants to the audience or stakeholders and to create a celebratory closure of the research study. One of the strengths of the present study is the adaptation of the photovoice method. The undergraduate students find the photovoice method motivating, efficient and an insightful and inspiring tool to express themselves and their views of food security and access to healthy food within their own communities, and this method has a greater impact than more traditional research methods^(23)^. Moreover, the combined use of photos, interviews and focus groups for data collection promotes data quality, credibility of findings and it makes the process more participatory in nature. On the other hand, such a combined approach for data collection makes the entire process more time-consuming, with a significant impact on timely reporting commitments. In addition, the fact that the photographs were taken during the COVID-19 pandemic meant that the students were not able to fully document the influence of face-to-face classes at university campuses, compared with online learning, on their food security and their access to healthy food.

To sum up, the present study provides important insights into undergraduate nursing and medical students’ perceptions of food security and their access to healthy food in Qatar. It also offers a venue for discussion around the circumstances that affect university students’ access to healthy food in Qatar and their sense of comfort around relying on fast foods during stressful times. Further research that is derived from participatory action research methods is required to foster university students’ engagement and to call for social change in terms of food access, quality, equity and security.
